# Evaluating the usefulness of VGI from Waze for the reporting of flash floods

**DOI:** 10.1038/s41598-022-08751-7

**Published:** 2022-03-28

**Authors:** Chris Lowrie, Andrew Kruczkiewicz, Shanna N. McClain, Miriam Nielsen, Simon J. Mason

**Affiliations:** 1 International Research Institute for Climate and Society, Climate School, Columbia University, New York, USA; 2 Coastal Resilience Lab, University of California Santa Cruz, Santa Cruz, CA USA; 3 National Aeronautics and Space Administration, Washington, DC USA; 4 Department of Earth and Environmental Science, Columbia University, New York, USA; 5 Red Cross Red Crescent Climate Centre, The Hague, the Netherlands; 6 Faculty of Geo-Information Science and Earth Observation, University of Twente, 7514 Enschede, AE the Netherlands; 7 National Aeronautics and Space Administration Goddard Institute for Space Studies, New York, NY USA

**Keywords:** Environmental social sciences, Natural hazards, Mathematics and computing

## Abstract

Using volunteered geographic information (VGI) to supplement disaster risk management systems, including forecasting, risk assessment, and disaster recovery, is increasingly popular. This attention is driven by difficulties in detection and characterization of hazards, as well as the rise of VGI appropriate for characterizing specific forms of risk. Flash-flood historical records, especially those that are impact-based, are not comprehensive, leading to additional barriers for flash-flood research and applications. In this paper we develop a method for associating VGI flood reporting clusters against authoritative data. Using Hurricane Harvey as a case study, VGI reports are assimilated into a spatial analytic framework that derives spatial and temporal clustering parameters supported by associations between Waze’s community-driven emergency operations center and authoritative reports. These parameters are then applied to find previously unreported likely flash flood-events. This study improves the understanding of the distribution of flash flooding during Hurricane Harvey and shows potential application to events in other areas where Waze data and reporting from official sources, such as the National Weather Service, are available.

## Introduction

### Flash flood warning procedures in the United States

In the United States, the National Weather Service (NWS) mandate requires warnings for a variety of severe weather types, including flash floods. Meteorologists at local NWS Weather Forecast Offices develop standard operating procedures for warning issuance based on understanding of the area, potential socioeconomic impacts and available meteorological data^1–3^. Warnings for flash floods are issued as geographic ‘storm-based’ (as opposed to county-based) polygons with a time duration, typically on the order of 3–10 h, and can cover as much as several hundred square kilometers or as little as 10 s of square kilometers^4^. During the warning duration, or soon after it expires, the forecast-office staff undertakes a confirmation process to verify whether or not a flash flood is taking or has taken place within the issued warning extent^5^. While the intent is to assess temporal overlap of any flash-flood warning with any potential flash flood^6^, there can be significant challenges in doing so with a high level of confidence, due in large part to the sparseness of sensors relative to the spatial and temporal scales of flash floods^7,8^. In addition to sensors, confirmations of a flash flood are derived from reports gathered primarily from specialists—trained storm watchers, law enforcement, and emergency management—but also from social media, local businesses, and news coverage^2,9,10^. If a flash-flood event is confirmed, the NWS Weather Forecast Office that issued the warning creates a Local Storm Report (LSR) containing a spatial–temporal point. LSRs are intended to be issued during the duration of the storm, to inform risk mitigation and response actions. Typically, a single confirmed report within the extent of the warning polygon is considered sufficient for verification, which speaks to the incentive to verify the warning, however this presents challenges in delineating the spatial extent and estimating the full impact of a flash flood^11^. After any subsequent meteorological phenomena pass, the Storm Data publication^12^ is created, which is intended to be the final source of truth for historical records.

Relative to other natural hazards in the US, there is a paucity in observational data for flash floods^13^. Compared to other flood types, historical records for flash floods are more likely to have significant gaps^14,15^. However, recent advances in unified flood and flash-flood data systems highlight the need for additional data sources to supplement reporting systems^16^. In the United States in 2005, only 46% of flash-flood warnings were verified^17^. Of the 54% of warnings that went unverified, it is unclear how many were false positives and how many coincided with actual, but unreported, flash-flood events.

For hurricane-related hazard risk, improving flood and flash-flood resilience is critical to building resilience^18,19^. For example, for Hurricane Harvey, all but 3 of the 68 direct fatalities were associated with freshwater drowning, with additional fatalities associated with motor-vehicle crashes and isolation from necessary medical services^20^, which could be indirectly attributed to flooding. A study of 62 hurricanes that made landfall in the United States found that the leading cause of death was freshwater drowning^21^, with over half of all flood-related deaths occurring in vehicles^22^. Taken together, these findings point to a need to improve flash-flood warning systems, particularly for mobility-related risk, in order to improve hurricane preparedness and resilience. This should be done for landfalling as well as near-coastal cyclones, given the possibility for intense rainfall related to these types of storms to be spatially distant from the storm center^23,24^. Considering options for event reporting beyond the conventional government and traditional media sources will help us expand and improve flash-flood warning systems where they are needed most^25–29^.

### VGI and citizen science for disaster risk management

VGI encompasses a wide variety of data sources created by platform users’ participatory actions, typically from mobile applications, that allow users to georeference an in-app action such as a social media post, possibly including text, photos or other media, attribution, and metadata^28^. In the last two decades this type of information has shown significant potential as a cost-effective source of geographic data, including in a wide variety of disaster management contexts including heat waves^29^, drought^30^, riverine and coastal floods^31–34^, and the assessment of shelter availability^35^. For floods in particular, VGI has been used for event detection, information dissemination, and post-event analysis^33^. One important benefit of VGI for disaster management is the ability to support decision making in near-real time situation analysis, referring to the properties of particular VGI applications that allow for real-time data gathering^34,36,37^. Incorporating VGI into disaster-risk reduction (DRR) has created opportunities to increase local participation and promote the integration of local knowledge in the DRR process^38^.

In operational contexts related to severe weather, VGI and mobile data is already in use. The NWS allows users to report information about storms through an online portal, Facebook, and Twitter^39^. The U.S. Department of Transportation Federal Highway Association uses traffic flow data, provided by INRIX, a provider of Location-Based Services data, to supplement real-time road sensors and aid situational awareness^40^. NOAA and the NWS gather reporting from the mobile application mPING^41^, which is used to supplement existing storm monitoring capabilities. Additionally, individual Weather Forecast Offices and news agencies may monitor social media for pertinent geographic information, although little evidence suggests this is standardized across all offices. The risks of VGI use, both the imprecise event descriptions and reporting guidelines, can be outweighed by the benefits of targeted use, especially in areas of limited data collection after disasters and/or with a lack of detailed event-attribution data^34,42^ and in areas with sharp gradients in socioeconomic conditions^43^. This is especially true for VGI sources that harness actively contributed data, as opposed to data that is passively harvested as a byproduct of unrelated user actions^38^. Data quality concerns should be addressed before assuming VGI integration will improve existing flash flood reporting capacity^44,45^.

Methods for VGI quality assurance include: the use of trained data validators; the creation of user credibility metrics and built-in incentives for accurate reporting; and statistical methods to refine and transform data^46^. Additionally, source applicability, semantic clarity, and meaningful descriptors of an event have been identified as an inherent aspect of VGI use^47,48^. Interoperability is also an important consideration in VGI methodology, with the intention that VGI data should supplement, rather than replace, traditional sources^47,49,50^. This focus on interoperability places focus on using VGI with analogous methodology to established reporting practices.

Geospatial methods for identifying meaningful information from VGI have included event clustering^34^, space–time statistics^51^, data mining to inherit properties from authoritative data sources^36^, cross-model validation^52,53^, and logistic regression^31^. VGI event clustering relative to authoritative sources is a core component of discerning meaningful information and source credibility^34^.

### Waze for disaster management

Compared to other social media applications, such as Twitter, Waze^54^ has seen less usage by the disaster management community. Waze is a mobile application for routing and turn-by-turn directions, with a substantial interface for users to provide feedback on real-time road conditions. While Twitter receives a large amount of attention, driven by its large user base, the site’s deficiencies are well documented. These deficiencies include semantic ambiguity that can arise from analyzing text-based posts^55,56^ and ambiguity in spatial and temporal data referencing^57^. Additionally, language-independence has been highlighted as a key consideration for VGI use^34^, which requires relevant metadata and attribution that is absent in Twitter. Waze, on the other hand, has a more limited usage domain—mobility—but is designed to provide maximum clarity for users within this domain. Given the value of Waze for traffic monitoring and pattern detection, and considering sufficient geographic accuracy, timely reporting and potential low latency and broad coverage, the crowdsourced data stream from Waze may be a valuable datasource for disaster risk management^58^, including for flood preparedness and response.

Acknowledging the value of supporting emergency management activities, the Waze community maintains The Waze Virtual Emergency Operations Center (VEOC) a volunteer-driven effort that monitors Waze reports, local weather stations, and official reporting services, and provides real-time support during storm events^59,60^. While the VEOC’s primary intention is to ‘keep the Waze map up-to-date’, the data is available for other analyses and decision making processes. The VEOC is a community effort related to and supported by Waze as a company, but volunteer-led. The VEOC data source adds another layer of validation on top of in-app reports and addresses many of the known requirements for VGI use, with data contributed by Waze users with a mostly high degree of experience. Waze user events have explicit tags to report flooding, addressing any semantic ambiguity that may arise from text-based VGI. Waze users have a credibility score and incentives for accurate reporting, increasing the likelihood that data are more accurate than other platforms. Waze reports are likely to exceed the spatial and temporal referencing of other sources, due to Waze’s built-in incentives for timely reporting and accurate information, designed to help the app provide local road conditions^61^. In the case of the VEOC, trained validators are reviewing flood reports from Waze users, as well as from local stations. The resulting dataset is focused on a particularly important set of storm risks—roadway flooding and flooding in areas that impact normal traffic flow conditions.

While Waze shows potential as a source of flood and flash-flood data, challenges remain. A primary concern is in understanding the scope of the Waze platform and data, particularly biases in the representativeness of its user base^38,62^. Despite large sample sizes, VGI from social media is rarely representative of the population^38,63^ and tends to overrepresent young and affluent citizens. Bias mitigation in VGI has emerged as a field, with important considerations for the use of VGI in decision making^62,64^. While VGI bias mitigation is not the focus of this research, it is an important consideration in the use of VGI to assist in disaster-risk reduction.

Waze is designed to detect roadway-related risks and impacts. Increased documentation of roadway-related flood events is a priority, given mobility disruptions leading to stressors in related social systems and services^65^. Further, with over half of all U.S flood-related drownings occurring within a vehicle^22^, improvement of in-transit flood reporting can lead to improved methods of tailored flash-flood warnings^66^ and potentially decrease indirect impacts of emergency response to other impacted areas^67,68^. Considering these findings, Waze is an excellent candidate for describing road-related flash-flood risk, but the current iteration of the product is unsuitable for non-roadway risk, or more specifically, non roadway floods sufficiently distant from roads as they would not impact traffic flow.

Waze flood reports communicate flood-related hazards to drivers based on the existence of water on the road with little information about severity. As such, user perception of flood risk likely varies widely.. While Waze users may be motivated to accurately report conditions, they may not view their warnings in the context of authoritative flood guidance. Previous studies have demonstrated the use of data aggregation in conjunction with comparisons to authoritative sources to establish credibility for a source of VGI^49,53,69^. The use of authoritative flash-flood reports along with geospatial clustering indicates a way forward for transforming Waze flood reports into meaningful information^34^, specifically the development of metrics to aggregate individual reports and alleviate biases and inconsistencies between users on any particular flood event. This paper focuses on the development of these magnitude validation metrics by creating a statistical methodology for associating Waze events to authoritative flash-flood reports such that clusters of Waze events can aid in flash-flood verification.

## Methods

### Methodology overview

This methodology establishes quantitative clustering parameters that meaningfully relate Waze flood report intensity to LSR occurrence. The methodology is driven by several assumptions. First, we assume that LSRs are related to Waze flood reports, that this relationship is not meaningful or is overly noisy when comparing individual reports, and that some unknown level of Waze intensity is appropriate for determining a relationship between the Waze reports and LSRs. Second, we assume that the LSR record is incomplete and that by deriving thresholds for clustering we may draw new useful information out of the Waze reports. By assuming LSR incompleteness and the potential for new Waze information, we seek to answer the following questions:

1. “Which LSRs are best supported by spatially and temporally dense Waze reports?”
2. “How spatially and temporally dense is Waze around these LSRs?”
3. “Are there locations of equivalent spatial and temporally Waze report density where LSRs are not present?”

### Data selection

**Figure 1:**
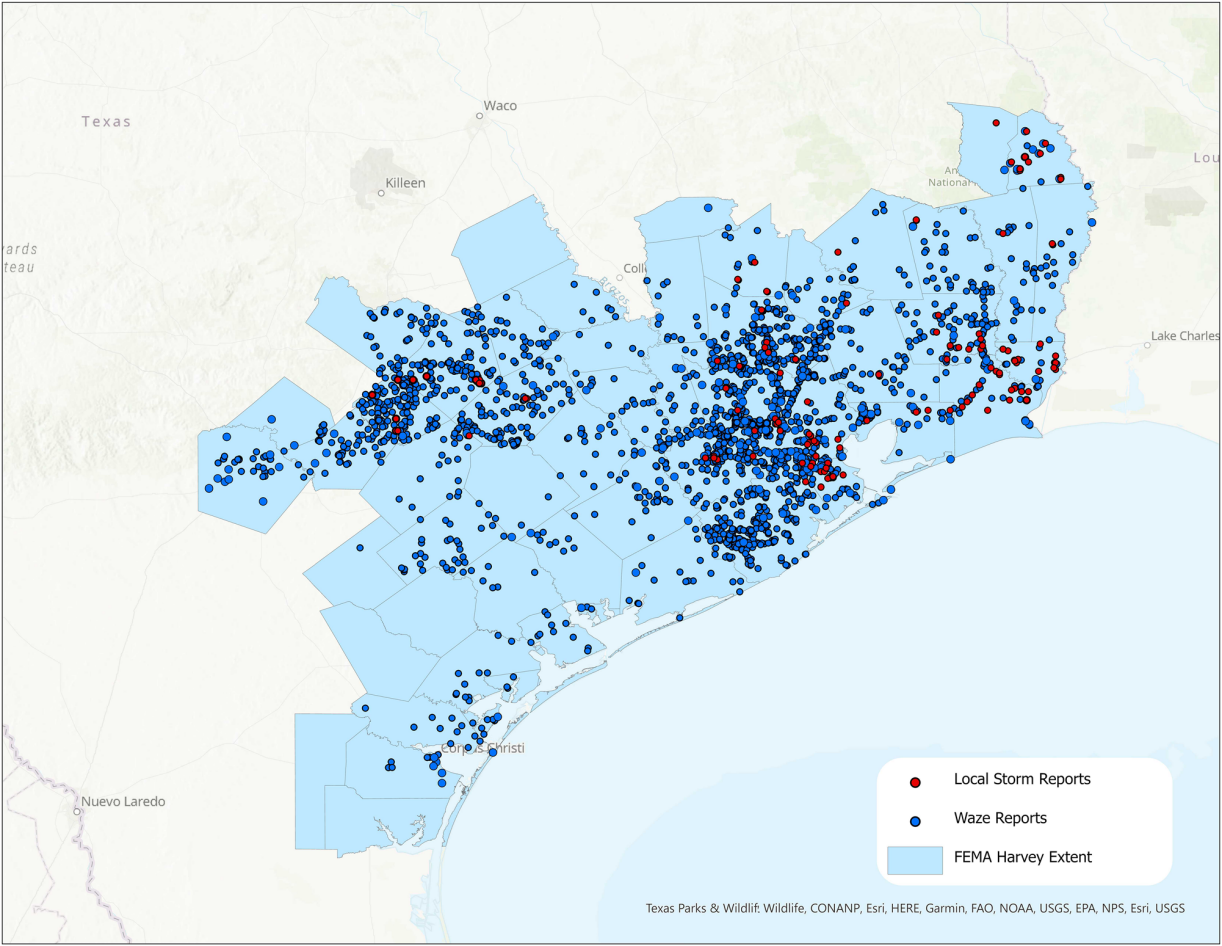
Hurricane Harvey's spatial extent covering 51 counties in South Texas. FEMA issued the storm's temporal extent as August 23–September 15, 2017^61^. Waze flood reports (blue) and Local Storm Reports (red) are overlaid on the FEMA extent. Map created using ArcGIS Pro^71^.

**Figure 2:**
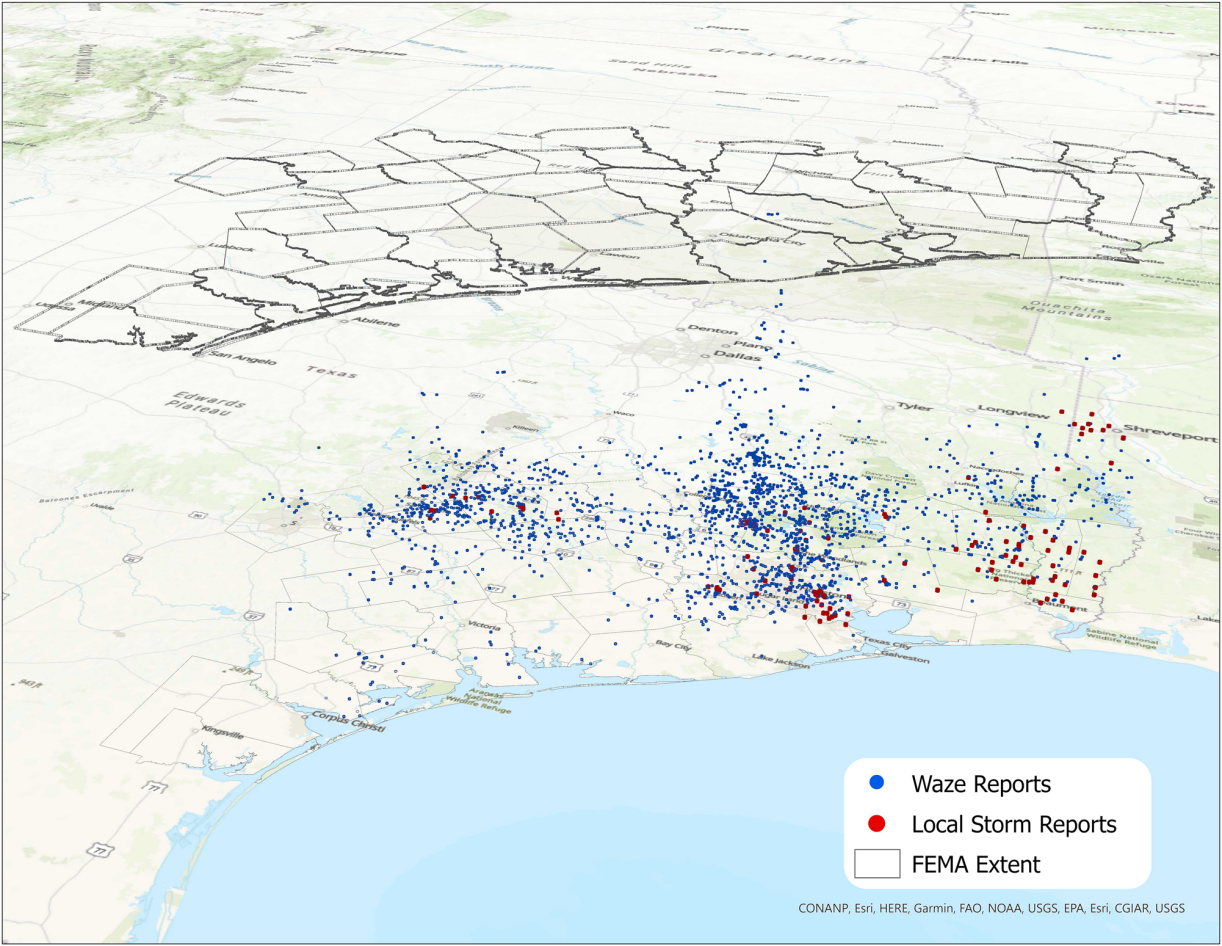
All Waze flood reports and Local Storm Reports. FEMA’s Hurricane Harvey extent is shown in grey, with the ground-referenced shapes representing reports occurring on August 23rd, and the vertical offset indicating time. The height of the floating county boundaries represent the latest Waze reports in the storm duration, September 12th. Map created using ArcGIS Pro^71^.

We extracted Waze flood reports from the Hurricane Harvey VEOC^59^. The Waze VEOC is available for many major storms in the United States. This effort involved processes for keeping road conditions up-to-date, such as verifying road conditions reported by users, as well as other crisis support functionality. The dataset consists of longitude, latitude, time (x, y, t) points, provided by Waze VEOC contributors, along with supplementary metadata denoting flooding. 2203 Waze reports were identified after filtering to the Hurricane Harvey boundary and time period as defined by FEMA^70^ (Figs. 1 and 2).

We downloaded LSRs from the Iowa Environmental Mesonet, which maintains a GIS server of hazards issued by the National Weather Service^72^. 125 LSRs were used after filtering for the FEMA extent and duration^70^. While the FEMA declaration persisted through September 12th, LSRs were not issued after August 31st, and Waze clusters were not detected after September 2nd.

The Storm Data publication was downloaded from NOAA’s National Centers for Environmental Information portal^11^. This file contained all storm points and was filtered to flash flood events during the duration and spatial extent of Hurricane Harvey.

### Statistical methodology

This methodology provides spatial and temporal clustering parameters that act as thresholds to validate clusters of Waze reports. Waze flood events were sequentially aggregated into spatial and temporal clusters around LSRs using bivariate k-nearest-neighbor clustering and spatial density analysis^73,74^. A specific number of Waze points (N) around a LSR denotes a cluster, and we derived a search radius around the LSR as the distance to include N points. Clustering was run for N = 10, 20, and 30 and calculations proceeded independently for each value of N, with the final results for each N-value feeding into a measure of robustness (described below).

**Figure 3:**
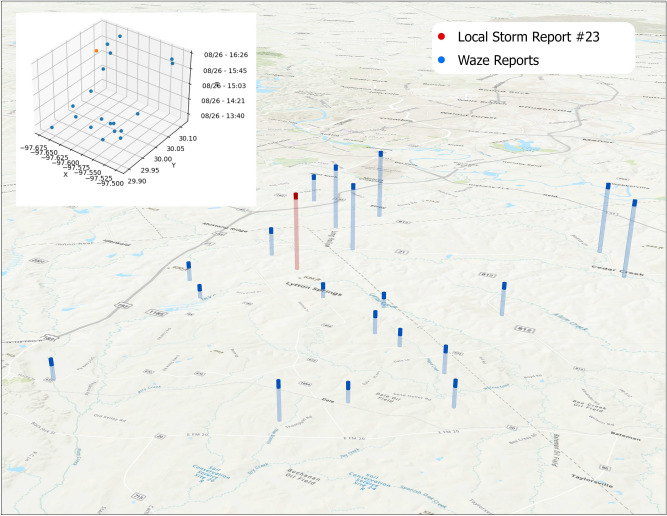
Example radius to include 20 points, for a single LSR. 20 Waze reports are included within 14 km over a time span of just over 3 h. The Local Storm Report (red) and Waze reports (blue) are shown, with height corresponding to time. The base of the bars shows the exact x,y location of the report, with taller bars occurring later in time. The difference between the tallest and shortest bars is approximately 6 h. The upper-left inset shows the same view with axis gridlines. Map created using ArcGIS Pro^71^.

By deriving a spatial distance for inclusion, and ordering these distances, a distribution was created of the clustering density. For a given value of N, a smaller radius indicates tighter clustering and higher density, indicating agreement between Waze reports and a LSR. Given the need to eliminate Waze reports that were unrelated temporally even if close spatially (i.e. points in the same location, but days or weeks apart), the Waze points available for inclusion were constrained using a time window around the LSR, of [−1, + 6] h (Waze reports up to one hour prior and 6 h after the LSR). This window aligns with NWS guidance on temporal evolution of flash floods and related impact^75^, as well as aligning with a sample of past Flash Flood Warnings of the NWS^2^, a visual inspection of lags between peaks in the Waze and LSR data, and feedback from the Waze VEOC community. To represent these data visually, a geographic space–time cube is used^76^. Figure 3 describes an example of tight Waze clustering around a single LSR for N = 10. Conceptually each LSR sits at the center of a cylinder, where the radius of the cylinder is the minimum distance required to include N points, and the height of the cylinder represents 7 h around the LSR.

The left tail of the spatial density distribution corresponds to the densest Waze reports, and choosing any percentile value on this distribution corresponds to choosing a spatial clustering threshold. Taken at the 5% level, each N-distribution yielded 7 LSRs and associated groups of Waze reports (125 LSRs * 0.05 = 6.25; results were rounded up). LSRs could be included as members of multiple N-groups, indicating that they were among the tightest clustered at multiple spatial scales.

The selected groups of high-spatial-density Waze reports were used in a top-hat kernel density estimate (bandwidth of ± 30 min) to further refine a temporal density threshold beyond the previously defined 7 h window. For each Waze point in a cluster, the number of neighboring points within 30 min was counted yielding a measure of temporal density. Table 1 describes an example temporal distance matrix for a single cluster of Waze points. From each temporal distribution, high clustering was again identified using the top 5% of reports. This extraction of temporally dense Waze reports completes the last step of a transformation from N, an arbitrary number of Waze reports to include, to spatial and temporal thresholds S and T, summarized by Table 2.

**Table 1:** Example temporal distance matrix for a group of highly clustered Waze events.

Waze report ID	707	720	722	723	733	746	790	805	1278	Total
707	0	7680	3480	1380	7080	7620	7980	3060	1740	3
720	7680	0	4200	6300	600	60	300	10,740	9420	4
722	3480	4200	0	2100	3600	4140	4500	6540	5220	1
723	1380	6300	2100	0	5700	6240	6600	4440	3120	2
733	7080	600	3600	5700	0	540	900	10,140	8820	4
746	7620	60	4140	6240	540	0	360	10,680	9360	4
790	7980	300	4500	6600	900	360	0	11,040	9720	4
805	3060	10,740	6540	4440	10,140	10,680	11,040	0	1320	2
1278	1740	9420	5220	3120	8820	9360	9720	1320	0	3

Values are in seconds. Totals are for counts of Waze reports within 1800 s.

**Table 2:** Spatial (S) and temporal (T) clustering values derived from N points.

N	S (m)	T (points within 30 min)
10	9275	8
20	16,636	15
30	26,017	21

Spatial and temporal clustering values derived for N points.

S and T were then applied to the full 2,203 Waze reports to filter events not meeting the spatial and temporal density thresholds. This created a set of reports for each value of N that are at least as dense as the top 5% densest reporting sets around the LSRs, but are no longer constrained to be near or related to an existing LSR. These density-supported reports will be referred to as Virtual Waze Reports, or VWRs.

The selected values of N are arbitrary and make determining a threshold challenging. Larger values of N describe clusters with more points and at larger spatial scales, as well as higher temporal density (given that more points are being included in the [−6, + 1] window). For this reason we derived a measure of robustness for each VWR by taking the number of supporting N-scales, ranging from 1 to 3 (Table 3). This measure of robustness across scales provides a conservative measure of value, showing reports that are verified at more than one scale.

**Table 3:** Robustness of Virtual Waze Reports. Robustness is measured on a scale of 1–3, based on the number of N-configurations that support a given VWR.

Number of virtual Waze reports supported by N-configurations	
Robustness	Number of VWRs
1	203
2	26
3	13

To identify if the Waze dataset provided new information, VWRs were de-duplicated against the existing LSRs. This de-duplication was done by removing VWRs that occurred within 17.6 km of an LSR within the same time period([−6, + 1] window). This number, 17.6 km, was calculated as the midpoint of the spatial clustering distances for N = 10 and N = 30. Several values were visually inspected as de-duplication criteria, and upon review, 17.6 km successfully isolated reports that could reasonably be seen as providing new information. Other thresholds could be used as well, depending on the level of conservatism desired in reporting VWRs. We did not find that this algorithm and dataset were sensitive to a deduplication number above 15 km. The 17.6 km de-duplication value is also larger, and thus more conservative than, the median nearest neighbor distance of the LSR dataset under the same − 6 h to + 1 h time constraints, which is about 14.25 km. This provides support to the idea that an LSR could have been issued at the identified VWRs, while providing new information and aligning with reasonable criteria for newness.

We also compared the remaining VWRs to the Storm Data publication, to see if any of the de-duplicated points were verified. As the number of VWRs was small, this was done by visual inspection in GIS. Inclusion in the Storm Data publication serves as further evidence that VWRs are detecting flash floods. The goal of this study is not to exceed, or even match, the Storm Data publication, as this is an eventual source of truth and does not necessarily provide any information on situational awareness during a storm. However, by eliminating any VWRs that coincide with LSRs, and showing that some or all of the remaining VWRs are contained in the Storm Data publication, we can show that VWRs are reporting actual flash floods and are improving situational awareness during a storm.

## Results

Table 2 describes the clustering parameters created during training of the algorithm on existing LSRs. These values describe the clustering of the tightest Waze reports around existing LSRs, at the 5% level. For example, 5% of LSRs have more than 10 Waze reports within 9275 m and the 5% highest density of those Waze reports is 8 reports within a 1 h time frame.

**Figure 4:**
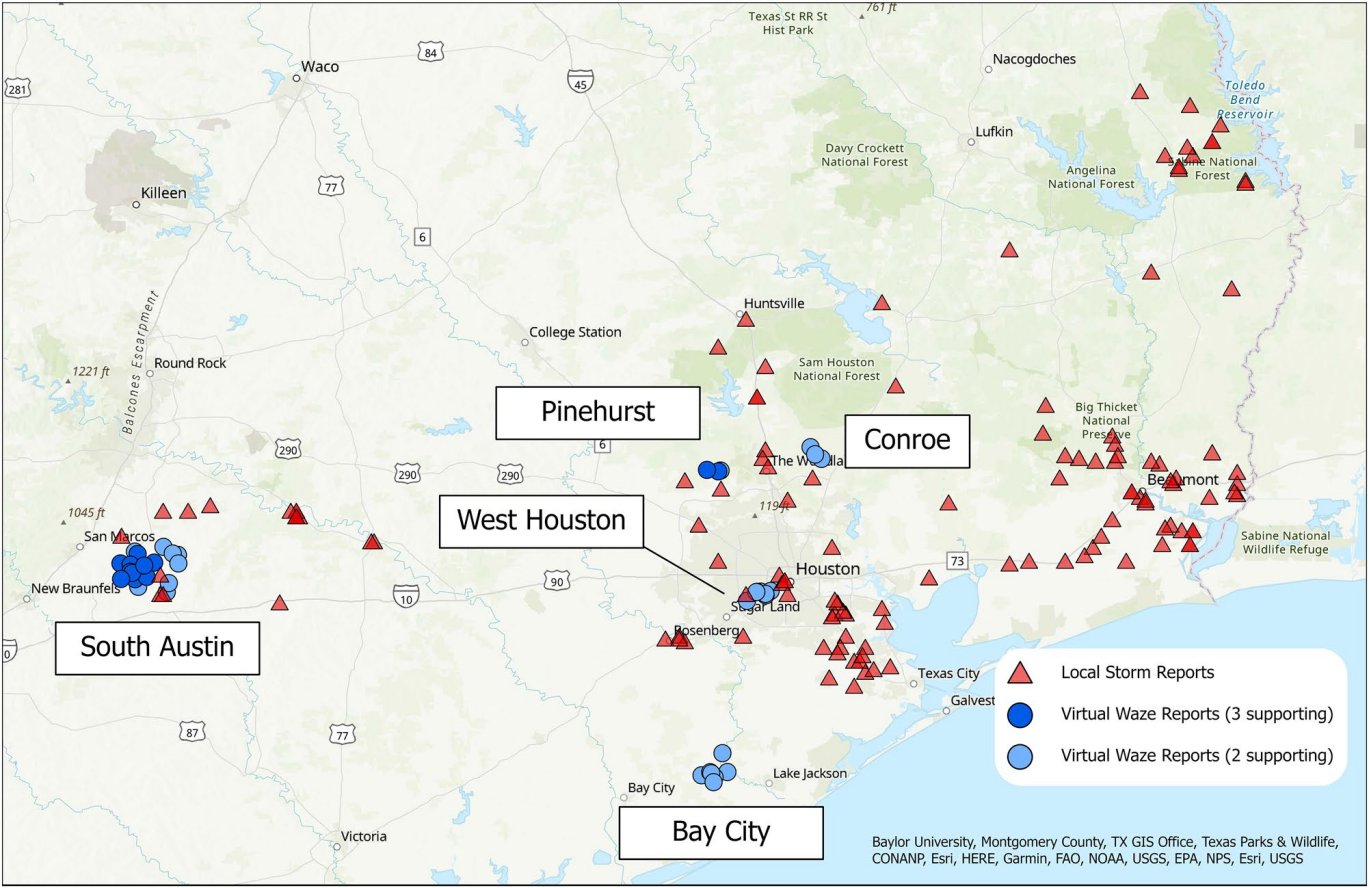
Remaining Virtual Waze Reports after de-duplication. LSRs are denoted by red triangles, VWRs supported by exactly 2 out of 3 configurations of N are shown in light blue, and VWRs supported by exactly 3 configurations are shown in dark blue. Note that while several new spatial clusters are apparent, the temporal dimension of flood reporting is not shown here. Clusters are labeled based on nearby population centers or points of interest. Map created using ArcGIS Pro^71^.

After clustering using the derived parameters, 13 VWRs were supported by 3 N-scales and 26 VWRs supported by 2 N-scales. These reports represent individual Waze reports that are supported by high spatial and temporal density at more than one scale, but may correspond to existing LSR locations and thus may not provide new information. After de-duplication, 11 VWRs are supported by 2 N-scales and 13 by 3 N-scales. Figure 4 describes the VWRs remaining after de-duplication in two dimensions, for VWRs supported by 2 and 3 N-configurations. Several new locations are spatially identifiable, particularly between Bay City and Grangerland near the coast, and to the south-west of Conroe in the north. Note that VWRs may appear in clusters, with several Waze reports meeting the criteria for promotion to a VWR. The number of nearby Waze reports does not necessarily address the severity or likelihood of a flash flood occurring and could be filtered or reduced via some form of clustering, but also does not inhibit the interpretation of the results. VWR clusters are labeled based on nearby population centers or points of interest.

**Figure 5:**
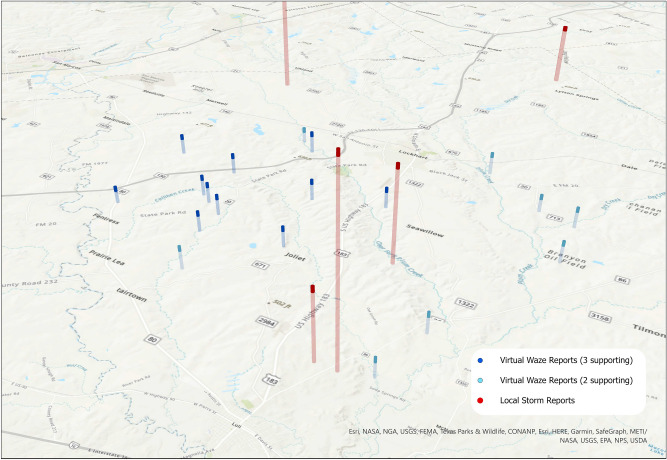
VWRs reported flooding to the South of Austin notably earlier than existing LSRs. The height of the bars represents time, with a difference of about 6.5 h between the VWRs (blue) and the LSR (red). In this example, VWRs appeared in the evening of the 26th, while the earliest LSRs were issued on the morning of the 27th. Map created using ArcGIS Pro^71^.

**Figure 6:**
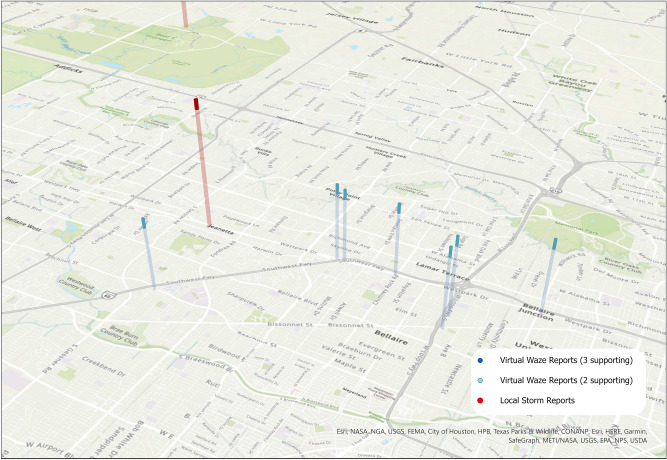
VWRs reported flooding west of Houston, notably earlier than existing LSRs. The height of the bars represents time, with a difference of about 6.5 h between the VWRs (blue) and the LSR (red). In this example, VWRs appeared in the evening of the 26th, while the LSR was issued on the morning of the 27th. Map created using ArcGIS Pro^71^.

In addition to denoting new spatial reporting areas, several VWRs occur in the same location as existing LSRs, but notably earlier, showing the capacity of the Waze data to respond to events in real-time. Figure 5 shows a cluster of VWRs near South Austin, which report flooding around 9 pm on the 26th, while LSRs were not issued until the morning of the 27th. Similar early reporting capabilities were demonstrated by VWRs directly to the west of Houston, near the junction of Interstates 69 and 610 (Fig. 6). While Figs. 5 and 6 are similar to Fig. 3, the VWRs reports provided information on flood conditions notably earlier than the reported LSRs.

Across N = 2 and N = 3, the VWRs detected form five new clusters of flash floods, either earlier than existing LSRs (West Houston and South Austin) or sufficiently far from existing LSRs (near Bay City, Conroe, and Pinehurst). For VWRs reported earlier than LSRs, the later existence of an LSR is considered as evidence that these reports are genuine and that they are providing new information in the form of earlier alerts. For VWRs that denote new spatial locations, two of the three were later included in the Storm Data publication (Bay City and Pinehurst), while the third (near Conroe) was not.

## Discussion

### Operational implications

The results show that VWRs are capable of detecting flash floods that align with LSR issuance and the Storm Data publication, and that VWRs are capable of providing real-time situational awareness by both near-real-time reporting, and by issuing reports that are both valid (as measured by inclusion in the Storm Data publication) and were not previously reported by LSR issuance. These are seen as compelling evidence that this form of VGI could be picked up in a semi-automated operational context to improve local awareness of flood risk during a storm event.

Our results indicate value in continuing to understand, and potentially integrate, certain well-vetted VGI data sources into the tools used for LSR issuance. While full automation of report issuance using the Waze VEOC is not recommended without understanding the source’s representativeness of the overall population, particularly socioeconomic biases and reporting equities^38,62,63,77^, it is clear that the Waze VEOC is providing timely and relevant information during a storm, and that clustering methods can be used to establish a signal for VGI intensity relative to an LSR baseline. This points to a semi-automated form of VGI contribution for report issuance, where Waze VEOC is monitored by a clustering algorithm, and VWRs are surfaced for WFOs to consider for issuance. Additionally, although the Waze VEOC serves a different overall purpose from NWS flash flood reporting, the two programs and any applicable trainings could be compared, with the goal of understanding and standardizing any overlap between the two processes. Finally, this data could be used within anticipatory action program development, which currently has seen little use in the context of flash floods and in urban areas. There have been few attempts to develop methods to identify which neighborhoods, far from the level of granularity of roads, are candidates for prioritization or deprioritization for anticipatory action when certain thresholds of flood risk are indicated within a forecast^78^.

### VGI applicability and representativeness

A critical next step in establishing the credibility of Waze is to understand the composure of the Waze sample and which groups and forms of risk may not be represented. Doing so allows for opportunities to understand the degree to which integration of such data into disaster management planning can have a negative, neutral, or positive influence on actions that have traditionally led to disproportionate impact on underserved populations and communities^79–81^. Additional calibration would aid this research as well, particularly by understanding the typical traffic cycle. While the research presented here has proposed a global clustering value, the natural next step is to understand the deviation from normal Waze reporting behavior, in addition to how Waze reporting volume compares to actual traffic volume, and the representation of drivers across socioeconomic status. Understanding the extent of this deviation would promote transparency in defining applicability of Waze data for representing flash flood risk in a more equitable manner, particularly if there are some populations such as older and non-white users that are underrepresented in the data, and if those communities’ movements are geographically biased. Using a longer-term Waze sample of an area would also help forecasters to understand the flood likelihood of a roadway as an input to flash flood warning issuance.

### Method portability

Methods are derived under the assumption of an incomplete record, which creates a challenge for training and evaluating VWR quality using many forms of statistical learning. The methods here are intended to operate on incomplete authoritative datasets, but also on datasets where there is reason to believe that the VGI source and the authoritative source will overlap incompletely, such as is the case of roadway flooding as a subset of all flash floods. Other forms of clustering and statistical learning may improve on the results presented here in the future, provided that the incomplete record is considered in the methods. The methods established here are simple, but could theoretically be extended to other VGI and authoritative sources, providing those sources meet well-established VGI criteria.

## Conclusion

This research demonstrates the use of spatial–temporal statistics to derive signals from authoritative data sources for using VGI in disaster awareness, opening up a new tool in the disaster management toolbox. Future research may focus on studying the applicability of metrics across storms, particularly if derived thresholds can be applied across geographies with varying topography, land-use, Waze adoption rates, and biases within the Waze sample. This research has not included Digital Elevation Models, atmospheric data, or streamflow gauges, all of which would be valuable additions for deriving a Waze signal. Including these data sources is an important next step towards determining the danger of a flood, for example via the rate of flow. The nature of the methods presented here—methods built entirely on point-to-point distance metrics—allows for portability across datasets, as well as the potential to expand the methods to include arbitrary model outputs and sensor data.

In the context of flood management, Waze is an ideal VGI candidate to complement existing flood observations and address a particularly dangerous form of flash flood risk. Additional research should be prioritized to understand the Waze sample, including user tendencies and sample biases. However, it is important to consider how future development of VGI processes may or may not increase representation of disaster impacts in underserved communities and that may reinforce existing disproportionalities. For next steps, we suggest prioritizing research on Waze representativeness and alignment with hydrological models to better describe flash flood onset and duration, extent, and impact.

## Data Availability

The data that support the findings of this study are available through Zenodo. DOI:10.5281/zenodo.5655741. Requests for future Waze VEOC events can be directed to the corresponding author.

## Code availability

The code that supports the findings of this study are available through Github:. Stable branch: https://github.com/lowriech/FlashFloodResponse/tree/code-submission-11_10_2021. Master branch (may include updates after the time of writing): https://github.com/lowriech/FlashFloodResponse.
