# Author Correction: Evaluating the usefulness of VGI from Waze for the reporting of flash floods

**DOI:** 10.1038/s41598-022-13306-x

**Published:** 2022-06-03

**Authors:** Chris Lowrie, Andrew Kruczkiewicz, Shanna N. McClain, Miriam Nielsen, Simon J. Mason

**Affiliations:** 1 International Research Institute for Climate and Society, Climate School, Columbia University, New York, USA; 2 Coastal Resilience Lab, University of California Santa Cruz, Santa Cruz, CA USA; 3 National Aeronautics and Space Administration, Washington, DC USA; 4 Department of Earth and Environmental Science, Columbia University, New York, USA; 5 Red Cross Red Crescent Climate Centre, The Hague, The Netherlands; 6 Faculty of Geo-Information Science and Earth Observation, University of Twente, 7514 Enschede, AE The Netherlands; 7 National Aeronautics and Space Administration Goddard Institute for Space Studies, New York, NY USA

Correction to: *Scientific Reports* 10.1038/s41598-022-08751-7, published online 28 March 2022

The original version of this Article omitted affiliations for Chris Lowrie, Andrew Krusckiewicz and Miriam Nielsen.

The correct affiliations for Chris Lowrie are listed below.

International Research Institute for Climate and Society, Climate School, Columbia University, New York, USA.

Coastal Resilience Lab, University of California Santa Cruz, Santa Cruz, CA, USA.

The correct affiliations for Andrew Krusckiewicz are listed below.

International Research Institute for Climate and Society, Climate School, Columbia University, New York, USA.

Red Cross Red Crescent Climate Centre, The Hague, The Netherlands.

Faculty of Geo-Information Science and Earth Observation, University of Twente, 7514, Enschede, AE, The Netherlands.

The correct affiliations for Miriam Nielsen are listed below.

Department of Earth and Environmental Science, Columbia University, New York, USA.

National Aeronautics and Space Administration Goddard Institute for Space Studies, New York, NY, USA.

The original Article has been corrected.

